# A data article on E-supply chain benefits from supplier׳s perspective

**DOI:** 10.1016/j.dib.2018.11.086

**Published:** 2018-11-23

**Authors:** Mohamed Dawood Shamout, Malek Bakheet Elayan

**Affiliations:** a American University in the Emirates, College of Business Administration (COBA), Dubai, UAE; b Institute of Public Administration, Riyadh, Saudi Arabia

## Abstract

This data article provides numeric values about one of the most important sectors in Dubai, Dubai Central Fruit and Vegetable Market (DCM), which attracted world attention through its solid infrastructure and outstanding business environment. The dataset has been collected using a questionnaire obtained from the operational managers in the selected market, and several ethical considerations during the data collection process have been applied to assure the quality and credibility of the obtained data. A structural equation modeling (SEM) was applied using IBM SPSS AMOS. In this data article, several analysis techniques have been used. This dataset shows a positive relationship between Electronic Supply Chain Management (E-SCM) processes and Customers from supplier׳s perspective. Furthermore, the results show that the assumption of the mediation role of E-SCM benefits is not supported.

**Specifications table**

**Table:** 

Subject area	*Business, Management*
More specific subject area	*Supply Chain Management, E-supply chain, Customers satisfaction*
Type of data	*Table, figure*
How data was acquired	*Survey. Cross-sectional, SEM, AMOS*
Data format	*Analyzed, Descriptive and Statistical data*
Experimental factors	*Sample consisted of operational managers, A valid questionnaire was used to collect the data*
Experimental features	*A descriptive approach was used to analyze the data. The data obtained from the operational level and from the selected sample, which gives this data a merit comparing the datasets obtained from frontline employees as the sector of the dataset, classified one of the most important sectors.*
Data source location	*Dubai, United Arab Emirates*
Data accessibility	*Data is included in this article*
Related research article	“Shamout, M. D., & Emeagwali, O. L. (2016). Examining the impact of electronic supply chain management processes on customer satisfaction: A literature review. *Business & Economic Horizons*, *12*(1)”.

**Value of the data**

• This data can be used to describe the role of the E-SCM process and its relation to customers satisfaction in one of vital sectors (i.e. Dubai central market)
• The results obtained from the dataset can be used to show a positive relationship between E-SCM process and Customers. Furthermore, the data collected on the operational level enhances the importance of the dataset.
• The data can be used for training purposes for PhD or master students practicing in the same field as this data article.
• This data article provides a clear image that shows how the norms of work and culture is different from non-western and western work life.

## 1 Data

Several analytical approaches were applied in this data article; descriptive statistics, correlation and structural equation modeling. Five tables are reported in this data article for clarifying the dimension of the data. Table 1 shows the pre-stages for data checking before proceeding forward with the Structural model, which is the model fit indices [1].Table 2 shows reliability and validity of the data instruments, as recommended by [2]. Further, Table 3 presents the values of correlation matrix between dataset constructs [3]. Table 4, Table 5 show the main results of this data article by estimating the association among the data article variables, direct and indirect effect. For the purpose of the data article, the operational managers will be included as the respondent for the questionnaire. Since the total numbers of operational managers are 434. The data includes the entire sample and distributed 434 questionnaires for the whole population.

**Table 1:** Goodness fit for the measurement model.

Goodness-of-fit indices	Cut-off points
Chi-square (X^2^) = 1503.55	*df* = 390, p = .000,
GFI = 0.83	Values close to 1 equals to perfect fit (Tanaka & Huba, 1985)
NFI = 0.86	Values close to 1 equals to perfect fit (Bentler & Bonett, 1980)
IFI = 0.89	Values close to 1 equals to perfect fit (Bollen, 1989),
TLI = 0.87	Values close to 1 equals to perfect fit (Bentler & Bonett, 1980)
CFI = 0.89	Values close to 1 equals to perfect fit t (McDonald & Marsh, 1990)
RMR = 0.048	Values < .060 equals to perfect fit (Browne & Cudeck, 1993).
RMSEA = 0.078	Values < .080 equals to perfect fit (Browne & Cudeck, 1993).
PCLOSE = 0.000	Values < .05 are accepted (Browne & Cudeck, 1993)
CMIN/DF = 3.85	Values >1 and < 5 were accepted (Marsh & Hocevar, 1985)

Note: “df, degree of freedom; GFI, goodness-of-fit indices; NFI, Normed Fit Index; CFI, comparative fit index;

IFI, incremental fit index; TLI, Tucker-Lewis index; RMR, root mean square residual;

RMSEA, root mean square error of approximation; CMIN/DF, Relative Chi-square”.

**Table 2:** Measurement model convergent and discriminant validity.

Variables	*α*	CR	AVE
Customer relationship management	0.84	0.83	0.62
Customer service management	0.85	0.85	0.66
Demand management	0.88	0.86	0.68
E-Fulfilment	0.76	0.76	0.52
E-Procurement	0.84	0.88	0.71
Product development and commercialization	0.84	0.88	0.70
Reverse logistic	0.87	0.84	0.65
**E-SCM benefits**	**0.91**	**0.89**	**0.46**
*Information Sharing and Trust*	*0.81*	–	–
*Lead time reduction*	*0.87*	–	–
*Cost and quality of service*	*0.80*	–	–

Note: “α, Cronbach׳s alpha; CR, Composite reliability; AVE, Average Variance Extracted”.

**Table 3:** Means, standard deviations (SD), and correlations of data article variables.

Variables	1	2	3	4	5	6	7	8	9
1. Customer relationship management	–								
2. Customer service management	0.789**	–							
3. Demand management	0.490**	0.483**	–						
4. E-Fulfilment	0.533**	0.548**	0.709**	–					
5. E-Procurement	0.498**	0.467**	0.426**	0.661**	–				
6. Product development and commercialization	0.548**	0.518**	0.415**	0.638**	0.781**	–			
7. Reverse logistic	0.473**	0.395**	0.320**	0.407**	0.619**	0.729**	–		
8. E-SCM benefits	0.362**	0.449**	0.256**	0.384**	0.419**	0.531**	0.492**	–	
9. Customer satisfaction	−0.036	-0.004	−0.044	0.007	0.078	0.095	0.091	0.050	–
Mean	4.31	4.22	4.33	4.28	4.29	4.23	4.16	4.17	4.36
Standard deviation	0.76	0.86	0.69	0.75	0.67	0.79	0.80	0.64	0.70

Note: Composite scores for each variable were computed by averaging respective item scores.

** Correlations are significant at the .01 level

**Table 4:** Maximum likelihood estimates.

Independent variables	Dependent variables	Coefficient estimates	Standard error	t-Statistics	*p*
Customer relationship management	E-SCM benefits	−0.177	0.033	−4.457	***
Customer service management	E-SCM benefits	0.361	0.029	9.075	***
Demand management	E-SCM benefits	−0.063	0.036	−1.573	0.116
E-Fulfilment	E-SCM benefits	0.092	0.033	2.307	0.021**
E-Procurement	E-SCM benefits	−0.071	0.037	−1.781	0.075*
Product development & commercialization	E-SCM benefits	0.284	0.032	7.149	***
Reverse logistic	E-SCM benefits	0.263	0.031	6.619	***
E-SCM benefits	Customer satisfaction	0.049	0.054	1.001	0.317

* Significant at the *p* < 0.1 level (two-tailed).

** Significant at the *p* < 0.05 level (two-tailed).

*** Significant at the *p* < 0.01 level (two-tailed).

**Table 5:** Break down of total effect of the data model.

Independent variables	Dependent variables	Total effect	Direct effect	Indirect effect
Customer relationship management	E-SCM benefits	−0.177	−0.177	0.000
Customer service management	E-SCM benefits	0.361	0.361	0.000
Demand management	E-SCM benefits	−0.063	−0.063	0.000
E-Fulfilment	E-SCM benefits	0.092	0.092	0.000
E-Procurement	E-SCM benefits	−0.071	−0.071	0.000
Product development & commercialization	E-SCM benefits	0.284	0.284	0.000
Reverse logistic	E-SCM benefits	0.263	0.263	0.000
**Mediation analysis**				
Overall E-CSM processes	E-SCM benefits	0.534	0.534	0.000
Overall E-CSM processes	Customer satisfaction	0.034	0.011	**0.023[**−**0.024 to 0.085, *p* > .01]**
E-SCM benefits	Customer satisfaction	0.044	0.044	0.000

In addition, getting data from the operational level of the organization allows us to get more practical information, since this level classified as a hub between top management and the customers. To maximize the benefits for both readers and researchers for this data article, several analytical approaches applied: first, descriptive statistics related to the dataset and the data article sample; second, the dataset fits the requirements of the selected analysis techniques (i.e. SEM), as well as several tests for normality, reliability and validity of the data to achieve the requirements for conducting the analysis using AMOS software. Moreover, assessing the model fits the indices and checks their eligibility within the collected dataset (i.e. check Tables below). After that we proceeded with the correlation analysis, direct and indirect among dataset constructs. Before the data collection process start, several ethical codes have been selected as “ethical consideration procedures” by following Diener and Crandall׳s procedures [4],such as “Harm to Participants”, “Consent”, “Deception” and “Privacy”, these codes used widely in the management data field [5].

## 2 Experimental Design, Materials and Methods

Using IBM SPSS and AMOS version 21.0 program this data generated the descriptive and inferential statistics with the aid of frequency analysis. It estimated the measurement model using AMOS to conduct structural equation modeling (SEM) [7], [8].

Following Bagozzi and Yi [9] recommendations, the data variables were subjected to CFA to evaluate internal consistency (i.e., reliability with Cronbach׳s alpha [α] and composite reliability [CR]), and construct validity (i.e., convergent, discriminant, and nomological validity).

All measures were subjected to confirmatory factor analysis (CFA) to provide support for the issues of dimensionality, convergent and discriminant validity [10]. Data screening and purification was conducted by inspecting the results from CFA, which suggested deletion of some scale items as a result of cross and/or low factor loadings that is less than 0.50 [10], [11]. Two items from a sub-construct “Information sharing and Trust” under E-SCM benefits; and one each from customer relationship management and customer service management were eliminated. In total 3 scale items were eliminated. Because of the scale purification and eliminations of low loading items, the measurement model fit the data adequately.

The full measurement model loaded satisfactorily, and all the fits indices were within the acceptable range (See Table 1). The data seems to have a normal distribution based on the outcome. As noted earlier, to gauge the potential threats of CMV, a single factor model was tested, the one or single factor model provided poorer fits in comparison to the measurement model. These poor fits suggests the propensity that the dataset is infiltrated with CMV is very low and probably does not seems to have occur [6]. See Table 1.

The factor item loadings in the data article exceeded 0.50, ranging from 0.50 to 0.97 with t-values ranges from 7.228 to 22.394. The average variance extracted (AVE) by each latent variable was above 0.50, except that of E-SCM benefits that stood at 0.46. Discriminant validity was checked using Fornell and Larcker׳s [12] criterion. Further, composite reliability (CR) ranged from 0.76 to 0.89, all above the threshold of 0.60 [9]. Additional internal consistency check was done with Cronbach׳s alphas (α), which were all above the cutoff point of 0.70 (Hair et al., 1998). The AVE value 0.46 was not a major problem because Fornell and Larcker׳s [12] previously noted that if CR is greater than 0.60 [7]. The construct still shows evidence of validity internal consistency. The present outcome delineates evidence of convergent and discriminant validity for the proposed model constructs, meaning the researcher can go ahead with further analysis. See Table 1, Table 2.

Furthermore, correlation analysis was conducted. The estimated correlations between the variables is below 0.85 which does provide additional evidence of discriminant validity (Kline, 2005). Correlation coefficients of the present data show that customer relationship management has a significant and positive association with E-SCM benefits (*r* = .362, *p* < .001). Customer service management significantly and positively correlated with E-SCM benefits (*r* = 0.449, *p* < .001). Likewise, demand management was found to positively associate with E-SCM benefits (*r* = 0.256, *p* < .001).

In similar fashion, e-Fulfilment has a positive association with E-SCM benefits (*r* = 0.384, *p* < .001). E-procurement significantly and positively correlated with E-SCM benefits (*r* = 0.419, *p* < .001). A positive and significant correlation was uncovered between product development and commercialization, and E-SCM benefits linkage (*r* = 0.531, *p* < .001). Finally, a positive and significant correlation was uncovered between reverse logistic and E-SCM benefits chain (*r* = 0.492, *p* < .001). Overall E-SCM processes and E-SCM benefits did not influence customer satisfaction (*r* = 0.034, *p* > .10) (*r* = 0.050, *p* > .10) respectively. Please see Table 3 and 4 below.

## 3 Mediation analysis

Overall E-SCM benefits was hypothesized to mediate the relationship between Overall E-SCM processes and customer satisfaction from suppliers’ perspective. “To augment the evidence of the indirect effect, we bootstrapped the model to produce a bias-corrected confidence interval for the standardized parameter estimate as recommended by” [13], [14], utilizing a validation sample of (*n* = 2000). The outcome shows that the standardized indirect effect of overall E-SCM processes on customer satisfaction from suppliers’ perspective through overall E-SCM benefits was not significant .023 (*p* > .10, 95% confidence interval: -.024 – .085). Based on the current outcome we concluded that there is no mediation effect. Please see Table 5 below.
